# Roles of Joule heating and spin-orbit torques in the direct current induced magnetization reversal

**DOI:** 10.1038/s41598-018-31201-2

**Published:** 2018-08-28

**Authors:** Dong Li, Shiwei Chen, Yalu Zuo, Jijun Yun, Baoshan Cui, Kai Wu, Xiaobin Guo, Dezheng Yang, Jianbo Wang, Li Xi

**Affiliations:** 1 Key Laboratory for Magnetism and Magnetic Materials of Ministry of Education & School of Physical Science and Technology, Lanzhou University, Lanzhou, 730000 People’s Republic of China; 2 Research Institute of Materials Science, Shanxi Normal University, Linfen, 041004 People’s Republic of China

## Abstract

Current-induced magnetization reversal via spin-orbit torques (SOTs) has been intensively studied in heavy-metal/ferromagnetic-metal/oxide heterostructures due to its promising application in low-energy consumption logic and memory devices. Here, we systematically study the function of Joule heating and SOTs in the current-induced magnetization reversal using Pt/Co/SmO_x_ and Pt/Co/AlO_x_ structures with different perpendicular magnetic anisotropies (PMAs). The SOT-induced effective fields, anisotropy field, switching field and switching current density (*J*_*c*_) are characterized using electric transport measurements based on the anomalous Hall effect and polar magneto-optical Kerr effect (MOKE). The results show that the current-generated Joule heating plays an assisted role in the reversal process by reducing switching field and enhancing SOT efficiency. The out-of-plane component of the damping-like-SOT effective field is responsible for the magnetization reversal. The obtained *J*_*c*_ for Pt/Co/SmO_x_ and Pt/Co/AlO_x_ structures with similar spin Hall angles and different PMAs remains roughly constant, revealing that the coherent switching model cannot fully explain the current-induced magnetization reversal. In contrast, by observing the domain wall nucleation and expansion using MOKE and comparing the damping-like-SOT effective field and switching field, we conclude that the current-induced magnetization reversal is dominated by the depinning model and *J*_*c*_ also immensely relies on the depinning field.

## Introduction

Manipulating magnetization reversal in a perpendicularly magnetized heavy metal (HM)/ferromagnetic metal (FM) structure by current-induced spin-orbit torques (SOTs) has attracted considerable attention in recent years due to its potential application in high density and low energy dissipation storages compared with the conventional spin-transfer-torque (STT) devices^1–6^. The spin Hall effect (SHE)^4,7,8^ resulted from HMs with the strong spin-orbit coupling (SOC) and the interfacial Rashba effect^2,9–12^ due to the inversion asymmetry of the interface or crystalline structure are considered to be the major sources that can enable spin current generation and spin accumulation when passing an in-plane charge current in HMs. Therefore, the polarized spins can exert efficient torques on the adjacent FM layer to manipulate the magnetization reversal in a commonly studied HM/FM/insulator heterostructure with perpendicular magnetic anisotropy (PMA). SOTs are divided into two components, damping-like and field-like torques, which can be obtained by measuring the corresponding effective fields that can be considered as an equivalent magnetic field^13–15^. In particular, the magnetization reversal is realized mainly depending on the damping-like effective field which is proportional to the spin Hall angles (*θ*_*SH*_) of HMs. Here, *θ*_*SH*_ is defined as the ratio of the spin current to the charge current, which can mirror the SOT’s efficiency.

At present, lots of works are concentrated on enhancing the current-induced magnetization switching efficiency and reducing the critical current density for decreasing the energy consumption of future SOT-based spintronic devices. Looking for HMs, such as Pt^4,16–18^, *β*-Ta^3,18–24^, Hf^25^ and *β*-W^26,27^ with the large *θ*_*SH*_ and/or achieving the large effective *θ*_*SH*_ based on HM/FM/HM structures in which two HM layers show opposite signs of *θ*_*SH*_^14,28,29^ were carried out more recently. Besides, some reports also reveal that *θ*_*SH*_ could be tuned by varying the thickness of HM^15,30^, decorating the interface between HM and FM^16,31,32^, changing the crystallinity of HM^33^ and even involving oxygen in HM^34^. Most of these studies can be boiled down to strengthen the driving force for the magnetization reversal and thereby improve the switching efficiency with a lower switching current density. However, the obstruction term should also be taken into consideration. For instance, Liu *et al*.^4^ reported that the anisotropy field needs to be overcome in the current-induced magnetization reversal based on a macrospin model at 0 K. Other reports^35–37^ showed that the depinning field is an essential parameter based on a domain wall (DW) depinning model. As a consequence, it is imperative to investigate the obstruction terms in the switching and understand the switching mechanism as well as corresponding influencing factors. Especially, when a current passing through the metal layers, the simultaneously generated Joule heating may elevate the sample temperature and consequently has an influence on the magnetic properties of devices. For example, Joule heating can give rise to the reduction of the critical depinning field, which has been widely reported by the theoretical simulation and/or experimental measurement^38–40^ in STT-based devices. However, the influences of Joule heating on the depinning field and current-induced magnetization reversal in SOT-based devices were rarely reported and usually ignored^41,42^.

In this work, we investigate the roles of Joule heating and SOTs played in the current-induced magnetization reversal using Pt/Co/SmO_x_ and Pt/Co/AlO_x_ structures with different PMAs. The results show that the large direct current (dc) can dramatically decrease the depinning field (i.e. switching field) due to the current-induced Joule heating effect. However, when applying a periodic pulse current with a small duty ratio to eliminate the Joule heating effect, we find that the effect of the current-induced SOTs on the switching field is weak. More importantly, the obtained switching current density (*J*_*c*_) remains roughly constant for Pt/Co/SmO_x_ and Pt/Co/AlO_x_ structures with quite different PMAs and nearly similar SOTs, revealing that the coherent switching model cannot fully explain the reversal behavior in the current-induced magnetization reversal process. In contrast, the switching mechanism could be the DW nucleation and expansion based on a depinning model. When the out-of-plane component of the damping-like SOT induced effective field overcomes the pinning field, a full magnetization reversal is realized. Therefore, *J*_*c*_ not only depends on the damping-like SOT induced effective field, but also on the depinning field. In addition, the current-induced Joule heating, which behaves like the temperature, plays an assisted role in the magnetization reversal by decreasing depinning field and increasing SOT efficiency.

## Results

### The anomalous Hall resistance measurements

**Figure 1:**
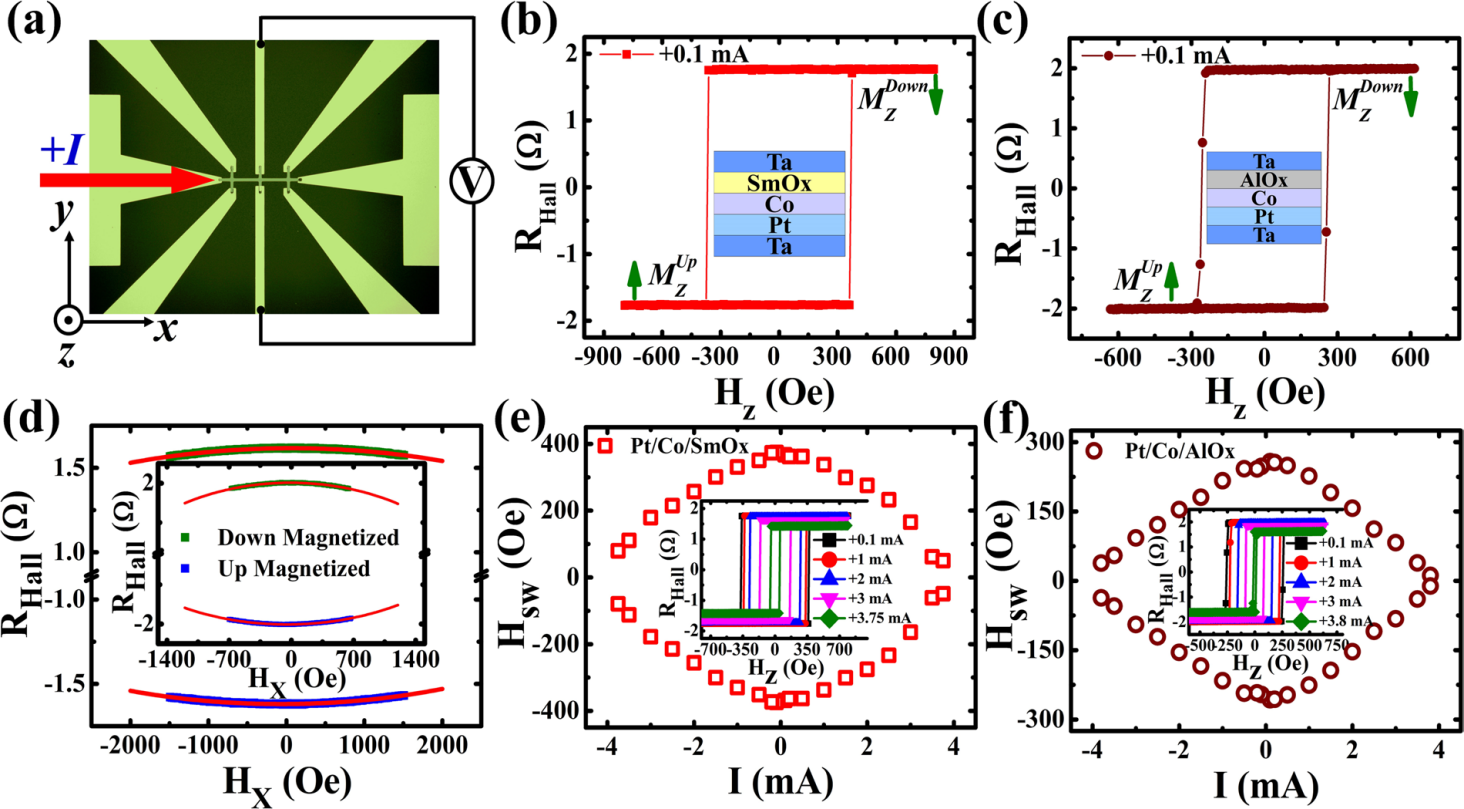
Device structure and anomalous Hall resistance measurements. (**a**) Optical images of the patterned Hall bar and measurement configuration. Dependence of *R*_*Hall*_ on *H*_*z*_ is measured at the current of +0.1 mA for Pt/Co/SmO_x_ (**b**) and Pt/Co/AlO_x_ (**c**) stacks. (**d**) *R*_*Hall*_ versus *H*_*x*_ for Pt/Co/SmO_x_ and the inset for Pt/Co/AlO_x_. The solid blue and green symbols correspond to “up” and “down” magnetized states, respectively. Red solid lines are the fitted curves. Switching phase diagram where *H*_*sw*_ varies against *I* for Pt/Co/SmO_x_ (**e**) and Pt/Co/AlO_x_ (**f**) stacks and the insets of them show the variation of *R*_*Hall*_-*H*_*z*_ loops at different currents.

Figure 1(a) schematically shows the patterned Hall bar structure and experimental configuration in which a direct current (*I*) is injected into the bar along the +*x* axis, and the voltage induced by the anomalous Hall effect (AHE) is measured along the *y* axis. Figure 1(b,c) show the anomalous Hall resistance (*R*_*Hall*_) against the external magnetic field along the *z* axis (*H*_*z*_) measured at a positive current of +0.1 mA (the current direction is illustrated in Fig. 1(a)) for Pt/Co/SmO_x_ and Pt/Co/AlO_x_, respectively. The square-shape-like loops demonstrate the presence of PMA. Since *R*_*Hall*_ is proportional to the perpendicular component of the magnetization (*M*_*z*_) of the Co layer, the magnetization direction ${M}_{Z}^{up}$ (${M}_{Z}^{down}$) corresponding to *R*_*Hall*_ < 0 (*R*_*Hall*_ > 0) can be identified in our experiment, which is labeled by green arrows in Fig. 1(b,c). The longitudinal resistance (*R*_*xx*_) is found to be around 6300 Ω and 3500 Ω for Pt/Co/SmO_x_ and Pt/Co/AlO_x_ bars with the length around 400 µm and 200 µm, respectively. As shown in Fig. 1(d), the anisotropy fields (*H*_*k*_) are determined by measuring *R*_*Hall*_ versus the in-plane magnetic field along the *x* axis (*H*_*x*_) at a small current of +0.1 mA using the formula^43,44^:

1 $${R}_{Hall}/{R}_{0}=\,\cos [\arcsin ({H}_{x}/{H}_{k})].$$

where *R*_0_ represents the Hall resistance when the magnetization is along the ***z*** direction. *H*_*k*_ is respectively estimated about 6650 ± 50 Oe and 2511 ± 11 Oe for Pt/Co/SmO_x_ and Pt/Co/AlO_x_ devices, which shows a large difference of the anisotropy field for both samples. Afterwards, we begin to investigate the dependence of the switching field (*H*_*sw*_) on *I*. *H*_*sw*_ is defined as an equivalent magnetic field which can make the moment from “up” state to “down” state or from “down” state to “up” state, which can be acquired from *R*_*Hall*_*-H*_*z*_ loops. *H*_*sw*_ as a function of *I* is displayed in Fig. 1(e,f) for Pt/Co/SmO_x_ and Pt/Co/AlO_x_, respectively, and the insets of Fig. 1(e,f) give representative *R*_*Hall*_*-H*_*z*_ loops at different *I*. One can see that *H*_*sw*_ decreases gradually as the current increases, which can be mainly ascribed to the increase of the Joule heating generated from direct currents (see Supplementary Material S1) instead of the current-induced damping-like-SOT effective field. Since the damping-like effective field is along the *x* direction and the value is not more than 15 Oe, it has almost no influence on the switching field, which will be discussed in details below.

### Polar Kerr hysteresis loops and Kerr differential images at different direct currents

**Figure 2:**
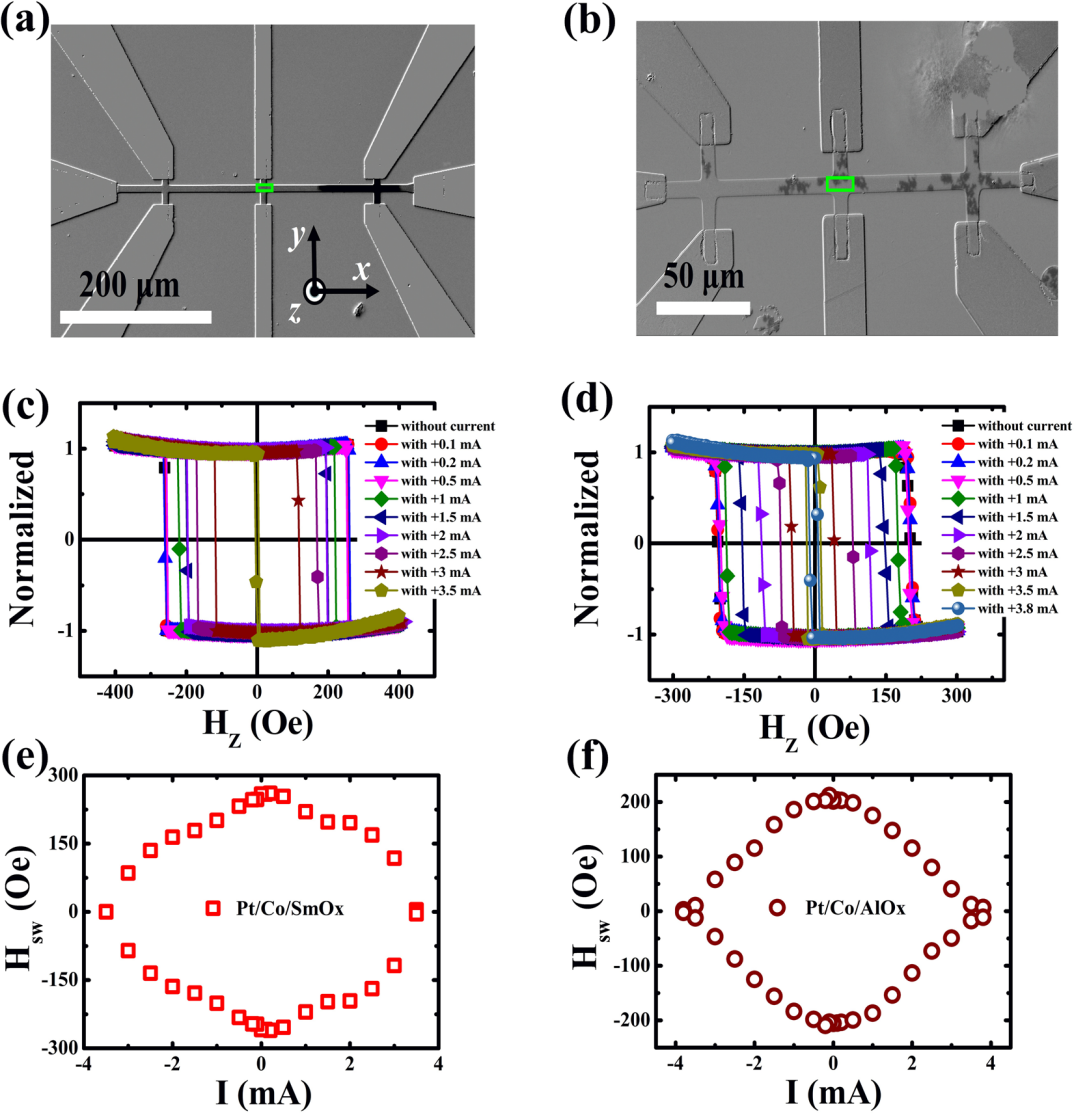
Polar Kerr hysteresis loops at different direct currents. Representative Kerr images for Pt/Co/SmO_x_ (**a**) and Pt/Co/AlO_x_ (**b**) during the field-induced magnetization switching, showing the differences of the DW nucleation. The light (deep) color shows the “up” (“down”) domain or magnetized state. The green rectangles stand for the measured regions where polar Kerr hysteresis loops are collected. Polar Kerr hysteresis loops for Pt/Co/SmO_x_ (**c**) and Pt/Co/AlO_x_ (**d**) stacks at different direct currents. Switching phase diagram where *H*_*sw*_ varies against *I* for Pt/Co/SmO_x_ (**e**) and Pt/Co/AlO_x_ (**f**) stacks.

Next, in order to reveal the effect of the direct current on the field-induced magnetization reversal, we used the polar magneto-optical Kerr microscopy technique^45^ to explore the polar Kerr hysteresis loops and Kerr differential images at different direct currents during the magnetization reversal. Figure 2(a,b) show the representative Kerr differential images at the nucleation state for Pt/Co/SmO_x_ and Pt/Co/AlO_x_ stacks, respectively. The light (deep) color in the images is defined as the “up” (“down”) domain or magnetized state. We find that the preferred nucleation sites are very different for the two stacks by applying an opposite *H*_*z*_ along the +*z* axis, starting from the saturated state using a large *H*_*z*_ along the −*z* axis. It can be ascribed to the different distributions of the depinning field due to the defects and impurities during the fabrication. For Pt/Co/SmO_x_ stacks, the nucleation site is relatively concentrated, showing an uniform DW expansion. However, the nucleation site is dispersive and then the DW at every nucleation site expands and further connects with each other to realize the magnetization reversal for Pt/Co/AlO_x_. Figure 2(c,d) show the polar Kerr hysteresis loops at different direct currents which were collected in the green rectangle regions displayed in Fig. 2(a,b). From Fig. 2(c,d), one can see that the loops gradually become narrow as the direct current increases for Pt/Co/SmO_x_ and Pt/Co/AlO_x_, implying the decrease of *H*_*sw*_ with the current increasing. In Fig. 2(e,f), we summarize the *I* dependence of *H*_*sw*_, which is well in agreement with that obtained using electric transport measurements based on AHE (see Fig. 1(e,f)). It means that the direct current indeed has an influence on *H*_*sw*_.

**Figure 3:**
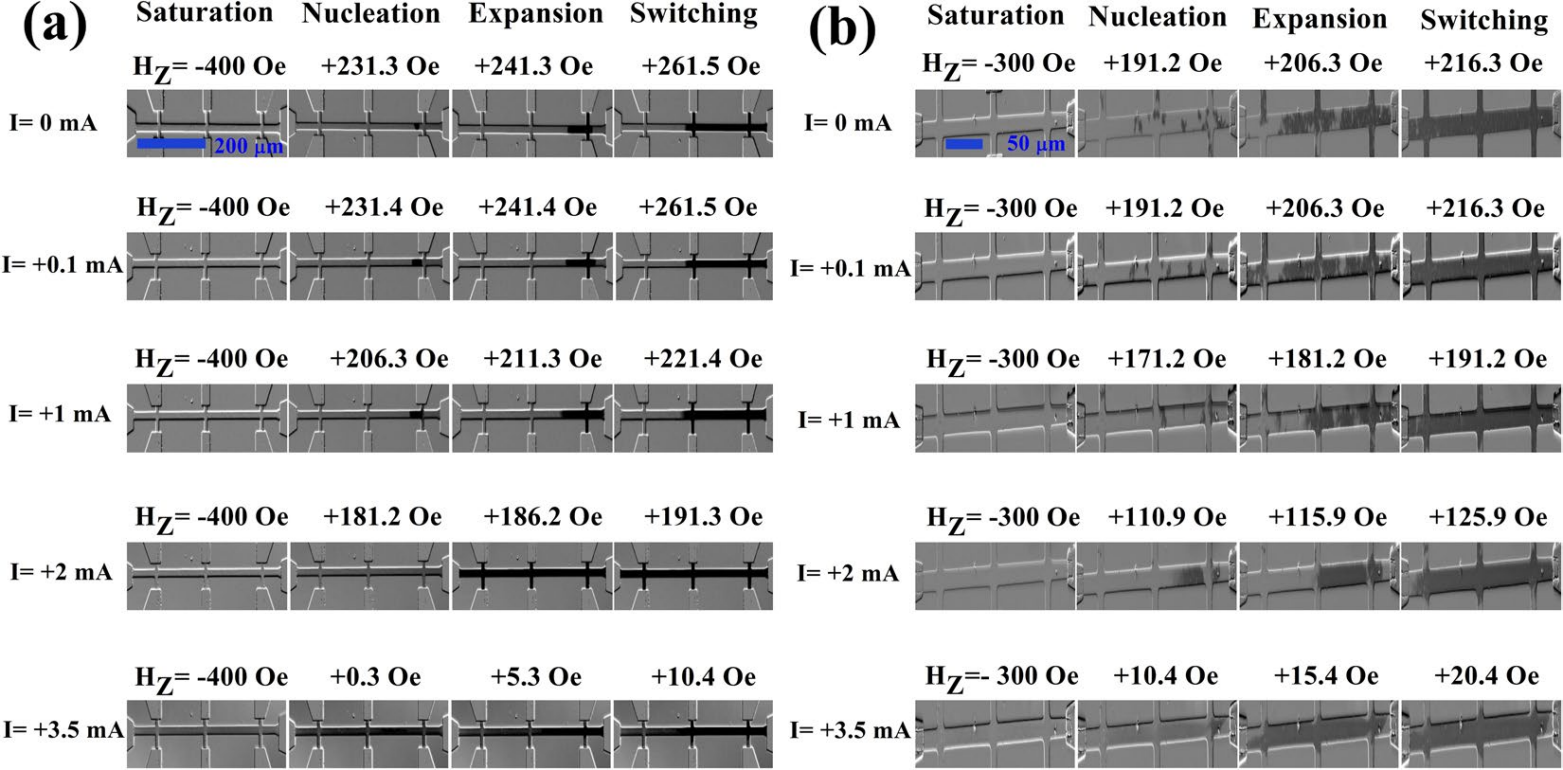
Kerr differential images of the DW nucleation and expansion at different direct currents. Representative Kerr images for Pt/Co/SmO_x_ (**a**) and Pt/Co/AlO_x_ (**b**) during the field-induced magnetization switching at various direct currents, showing the effect of the direct current on DW nucleation and expansion. The light (deep) color shows the “up” (“down”) domain or magnetized state.

When collecting the Kerr hysteresis loops, the images of the DW nucleation and expansion were also recorded. Figure 3(a,b) show the representative Kerr differential images of the DW nucleation and expansion at different direct currents for Pt/Co/SmO_x_ and Pt/Co/AlO_x_, respectively. At first, an “up” domain in the saturated state appears under a large *H*_*z*_ (−400 Oe for Pt/Co/SmO_x_ and −300 Oe for Pt/Co/AlO_x_). Afterwards, an opposite *H*_*z*_ is applied to form the opposite nucleation sites. And then these nucleation sites further expand as the *H*_*z*_ goes on increasing until to accomplish the magnetization reversal of the green-rectangle measured regions shown in Fig. 2(a,b). In Fig. 3(a,b), we can observe that both the nucleation field and switching field in the measured regions gradually decrease as the direct current increases for the two stacks. Particularly, the nucleation site and expansion speed evidently change at the large current, revealing that the direct current could affect the magnetization reversal by means of the DW nucleation and expansion.

### The influence of Joule heating on the switching field

**Figure 4:**
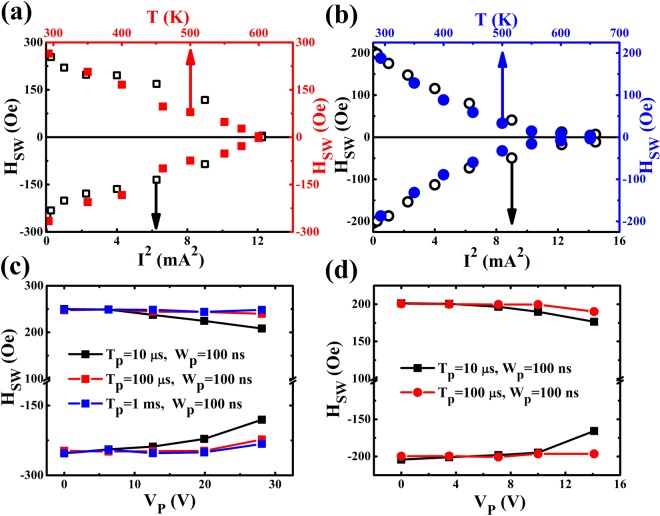
The direct current, temperature and pulse voltage dependences of the switching field. *H*_*sw*_ as a function of *I*^2^ and *T* for Pt/Co/SmO_x_ (**a**) and Pt/Co/AlO_x_ (**b**), showing the effect of the current-induced Joule heating. *H*_*sw*_ against *V*_*p*_ at various *T*_*p*_ for Pt/Co/SmO_x_ (**c**) and Pt/Co/AlO_x_ (**d**), showing the effect of the current-induced spin-orbit torques.

In order to distinguish the contributions of the Joule heating (*Q*_*J*_ ∝ *I*^2^) and current-induced SOT (*τ* ∝ *I*) to the field-induced magnetization reversal, we also measured the polar Kerr hysteresis loops and Kerr differential images at different temperatures (*T*) and pulse voltages (*V*_*p*_) (see Supplementary Materials S2 and S3) to investigate the effect of the *T* and *V*_*p*_ on *H*_*sw*_. In Fig. 4(a,b), we directly compare the *I*^2^ and *T* dependences of the *H*_*sw*_ for Pt/Co/SmO_x_ and Pt/Co/AlO_x_. The *H*_*sw*_*-I*^2^ is extracted from the polar Kerr hysteresis loops measured by varying *I* at room temperature (RT) (see Fig. 2(c,d)), while *H*_*sw*_*-T* is at various *T* without any currents (see Supplementary Material S2). From Fig. 4(a,b), one can see that the different dependences of the *H*_*sw*_ on *T* for Pt/Co/SmO_x_ and Pt/Co/AlO_x_, demonstrating the different domain wall nucleation and expansion behaviors (see Fig. 2(a,b)). However, the *I*^2^ and *T* dependences of the *H*_*sw*_ exhibit the same variation tendency, revealing that the Joule heating from the direct current should not be negligible and has a conclusive effect on *H*_*sw*_. A strong Joule heating effect existed in our samples may be ascribed to that the film stacks were deposited on a 500 μm thick glass substrate with the poor thermal conductivity. Torrejon *et al*.^46^ reported that even though a 2 ns wide current pulse with the amplitude of 10^8^ A/cm^2^ passing through a 400 × 20 nm^2^ FeNi nanostrip on Si substrate with the 100 nm thick SiO_2_ oxide layer, it has a 327 K temperature rise compared to a 53 K temperature rise on Si substrate with a 2 nm thick native oxide layer due to the poor thermal conductivity of SiO_2_ layer. In addition, we also summarize *H*_*sw*_ against *V*_*p*_ at different pulse periods (*T*_*p*_) with the fixed pulse width (*W*_*p*_) of 100 ns to study the effect of the current-induced SOT on *H*_*sw*_ by fading out the Joule heating effect, which is shown in Fig. 4(c,d) for Pt/Co/SmO_x_ and Pt/Co/AlO_x_, respectively. It is noted that the *V*_*p*_ of 28.1 V (14.1 V) corresponds to the pulse current (*I*_*p*_) of about 4.5 mA (4 mA) for Pt/Co/SmO_x_ (Pt/Co/AlO_x_). When *T*_*p*_ = 10 µs, *H*_*sw*_ decreases weakly as *V*_*p*_ increases and when *T*_*p*_ = 100 µs even 1 ms, *H*_*sw*_ nearly keeps constant. It means that the effect of current-induced SOT on *H*_*sw*_ is rather weak compared to the influence of the temperature or Joule heating on *H*_*sw*_. Furthermore, it is also demonstrated that the Joule heating becomes prominent as *T*_*p*_ decreases or the duty ratio of *W*_*p*_/*T*_*p*_ increases. Therefore, we can conclude that the decrease of *H*_*sw*_ with the direct current increasing should be ascribed to the increase of the direct current generated Joule heating rather than the current-induced SOT and the Joule heating like *T* can exert a considerable influence on *H*_*sw*_. In order to gain more insight on the function of the Joule heating and SOT in the current-induced magnetization reversal process, we quantitatively explore the relationships between *H*_*sw*_, SOT, and *J*_*c*_, which will be described in the next part.

### The harmonic Hall voltage measurements and current-induced effective fields

**Figure 5:**
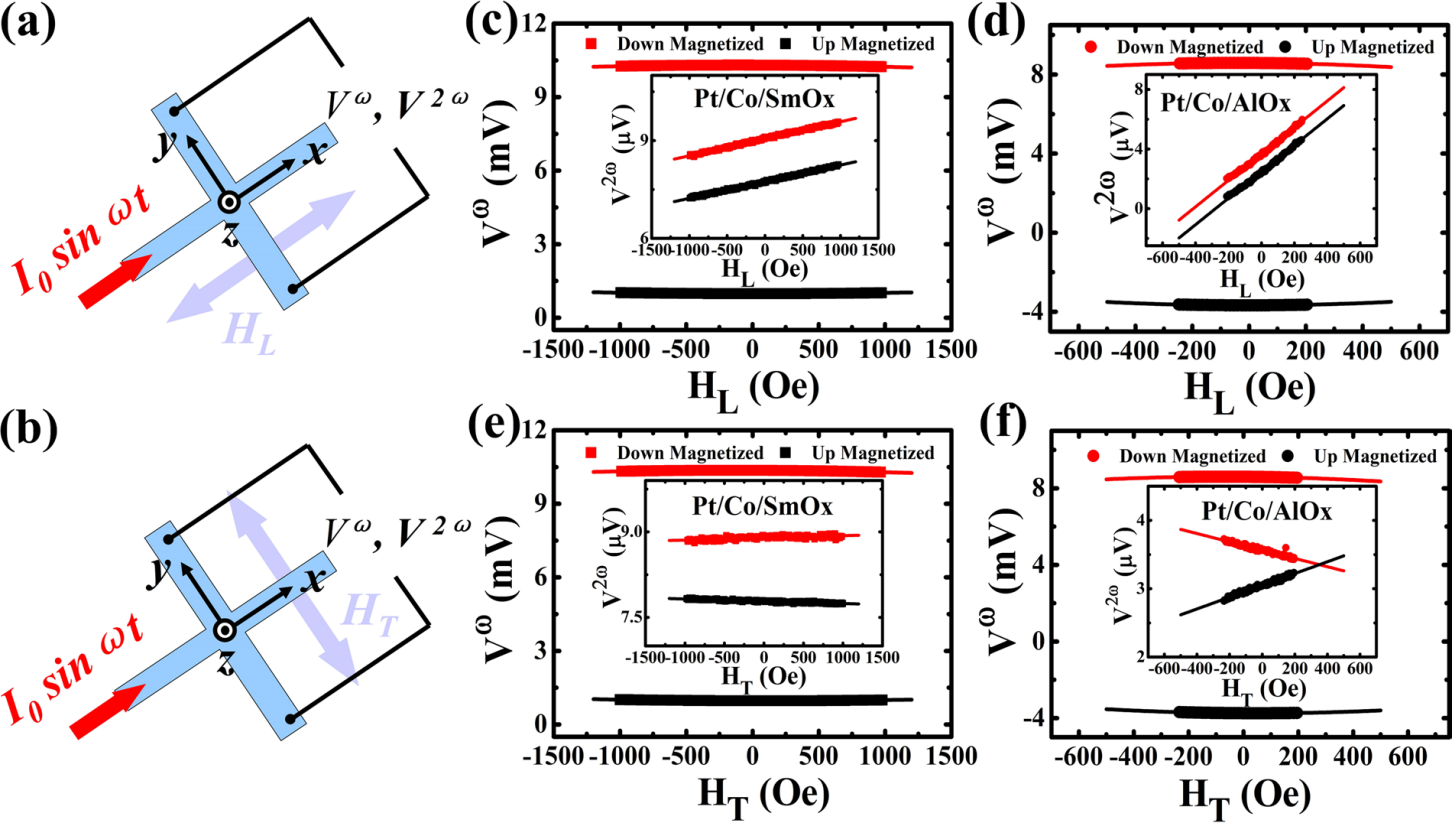
The harmonic Hall voltage measurements. Illustrations of the SOT-induced damping-like (**a**) and field-like (**b**) effective fields measurements. (**c**–**f**) *V*^*ω*^ as a function of the in-plane *H*_*L*_ and *H*_*T*_ external magnetic fields. The inset in each figure represents the *V*^*2ω*^ versus *H*_*L*_ and *H*_*T*_. The solid black and red symbols denote “up” and “down” magnetized states, respectively and the solid lines represent the linear and quadratic fitting curves.

The current-induced damping-like and field-like effective fields were quantified by the harmonic Hall voltage measurement technique. The measurement diagrams are depicted in Fig. 5(a,b). When passing a sinusoidal current through the Hall bars along *x* axis, the first (*V*^*ω*^) and second (*V*^*2ω*^) harmonic voltages were collected along *y* axis via an Analog-Digital (AD)/Digital-Analog (DA) data acquisition card. Therefore, the current-induced longitudinal damping-like effective field (*H*_*DL*_) and transverse field-like effective field (*H*_*FL*_) can be obtained by sweeping the external longitudinal magnetic field (*H*_*L*_) and transverse magnetic field (*H*_*T*_), respectively. And then the *H*_*DL*_ and *H*_*FL*_ are determined by the equations^19^:

2 $$\begin{array}{rcl}{H}_{DL} & = & -2\frac{\partial {V}^{2\omega }}{\partial {H}_{L}}/\frac{{\partial }^{2}{V}^{\omega }}{\partial {H}_{L}^{2}}\\ {H}_{FL} & = & -2\frac{\partial {V}^{2\omega }}{\partial {H}_{T}}/\frac{{\partial }^{2}{V}^{\omega }}{\partial {H}_{T}^{2}}.\end{array}$$

Figure 5(c–f)) represent the first harmonic voltages *V*^*ω*^ as a function of *H*_*L*_ (*H*_*T*_) for the Pt/Co/SmO_x_ and Pt/Co/AlO_x_ devices, respectively. The insets in each figure show the second harmonic voltages *V*^*2ω*^ against *H*_*L*_ and *H*_*T*_ for the corresponding devices. Furthermore, in order to calculate *H*_*DL*_ and *H*_*FL*_ according to the Eq. (2), the quadratic and linear function were used to fit the first and second harmonic curves, respectively. The results are shown in the Supplementary Material S4. It should be noted that the measured harmonic voltages generally contain the contributions resulted from both the anomalous Hall effect and planar Hall effect (PHE). Thus, a correction must be taken into consideration in Eq. (2) if these two contributions are comparable and the corrected effective fields can be expressed by^20,47–49^:

3 $${\rm{\Delta }}{H}_{DL(FL)}=\frac{{H}_{DL(FL)}\pm 2\xi {H}_{FL(DL)}}{1-4{\xi }^{2}}.$$

where the ± sign refers to the “up” and “down” magnetized states and *ξ* is the ratio of the planar Hall resistance (Δ*R*_*PHE*_) to anomalous Hall resistance (Δ*R*_*AHE*_). We measure the Δ*R*_*PHE*_ and Δ*R*_*AHE*_ (see Supplementary Material S4 for details about PHE measurements) and the ratio *ξ* ~ 0.15 (*ξ* = Δ*R*_*PHE*_/Δ*R*_*AHE*_) for Pt/Co/SmO_x_ and Pt/Co/AlO_x_ is calculated. It means that the PHE correction is not negligible and should be considered.

**Figure 6:**
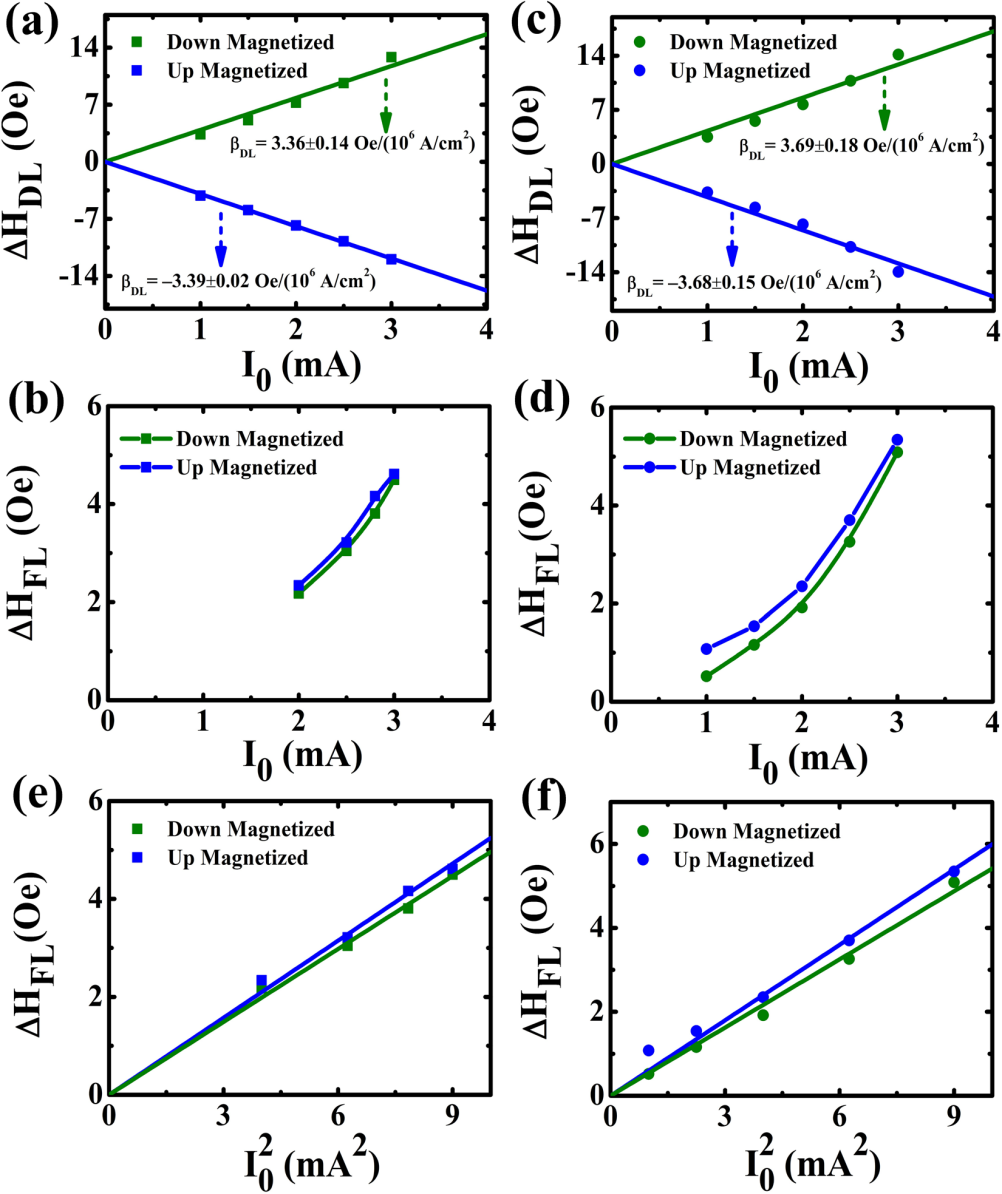
Current-induced effective fields after the planar Hall effect correction. (**a**–**d**) SOT-induced damping-like and field-like effective fields for Pt/Co/SmO_x_ (Pt/Co/AlO_x_) as a function of the amplitude *I*_0_ of the sinusoidal current. The blue and green symbols denote “up” and “down” magnetized states, respectively. The field-like effective fields against ${I}_{0}^{2}$ for Pt/Co/SmO_x_ (**e**) and Pt/Co/AlO_x_ (**f**), respectively.

The corrected damping-like effective fields (Δ*H*_*DL*_) as a function of the amplitude (*I*_0_) of the sinusoidal current for both devices are plotted in Fig. 6(a and c). The roughly linear dependence of the effective fields on *I*_0_ indicates that the contributions of the non-linear effects including the current-induced Joule heating can be weak at lower currents compared to the current-induced damping-like effective fields. From the linear relationship, the damping-like SOT efficiency (Δ*β*_*DL*_) defined by Δ*β*_*DL*_ = Δ*H*_*DL*_*/J*_*e*_ (*J*_*e*_ is the charge current density) is found to be −3.39 ± 0.02 Oe/(10^6^ A/cm^2^) (3.36 ± 0.14 Oe/(10^6^ A/cm^2^)) for the “up” (“down”) magnetized states for Pt/Co/SmO_x_ and −3.68 ± 0.15 Oe/(10^6^ A/cm^2^) (3.69 ± 0.18 Oe/(10^6^ A/cm^2^)) for the “up” (“down”) magnetized states for Pt/Co/AlO_x_. Obviously, the damping-like-SOT-induced effective fields are almost identical for two samples, which is due to the same contribution from the heavy metal Pt. Meanwhile, according to the formula^4,50,51^:

4 $${\theta }_{SH}={J}_{s}/{J}_{e}=(\frac{2|e|{M}_{s}{t}_{FM}}{\hslash })({\rm{\Delta }}{H}_{DL}/{J}_{e}).$$

where *e* is the elementary charge, *M*_*s*_ is the saturation magnetization of the samples which is determined to be about 1.15 × 10^6^ A/m and 1.12 × 10^6^ A/m at RT for Pt/Co/SmO_x_ and Pt/Co/AlO_x_, respectively, *t*_*FM*_ is the thickness of the cobalt layer and *ħ* is the reduced Planck constant. We can also estimate the *θ*_*SH*_ at RT which equals to 0.071 ± 0.002 and 0.074 ± 0.004 for Pt/Co/SmO_x_ and Pt/Co/AlO_x_, respectively, close to the previous reported value for Pt^52,53^.

The corrected field-like effective fields (Δ*H*_*FL*_) are also plotted as a function of *I*_0_ in the same manner for both devices displayed in Fig. 6(b and d). Unlike Δ*H*_*DL*_, a non-linear quadratic behavior of Δ*H*_*FL*_ versus *I*_0_ is observed. It is not well in agreement with the linear dependence of the field-like effective fields on the currents reported in the references^7,54,55^. One possible explanation for the non-linear behavior is the Joule heating effect. Although the thermal effect also affects Δ*H*_*DL*_ at the same time, it seems to be weak as discussed above. It could be ascribed to the different temperature dependences of Δ*H*_*DL*_*/J*_*e*_ and Δ*H*_*FL*_*/J*_*e*_ as reported in the references^20,56,57^, in which Δ*H*_*FL*_*/J*_*e*_ is highly sensitive to temperature, while Δ*H*_*DL*_*/J*_*e*_ shows a weak temperature dependence in Ta-based, Pt-based, and W-based samples. In order to demonstrate the effect of the Joule heating, we extract the dependence of Δ*H*_*FL*_ on ${I}_{0}^{2}$ shown in Fig. 6(e,f) for Pt/Co/SmO_x_ and Pt/Co/AlO_x_, respectively. From Fig. 6(e,f), one can see that the Δ*H*_*FL*_ varies linearly with ${I}_{0}^{2}$, implying that the contribution of the Joule heating cannot be negligible compared to the current-induced field-like effective fields from SHE or interfacial Rashba effect.

## Discussion

**Figure 7:**
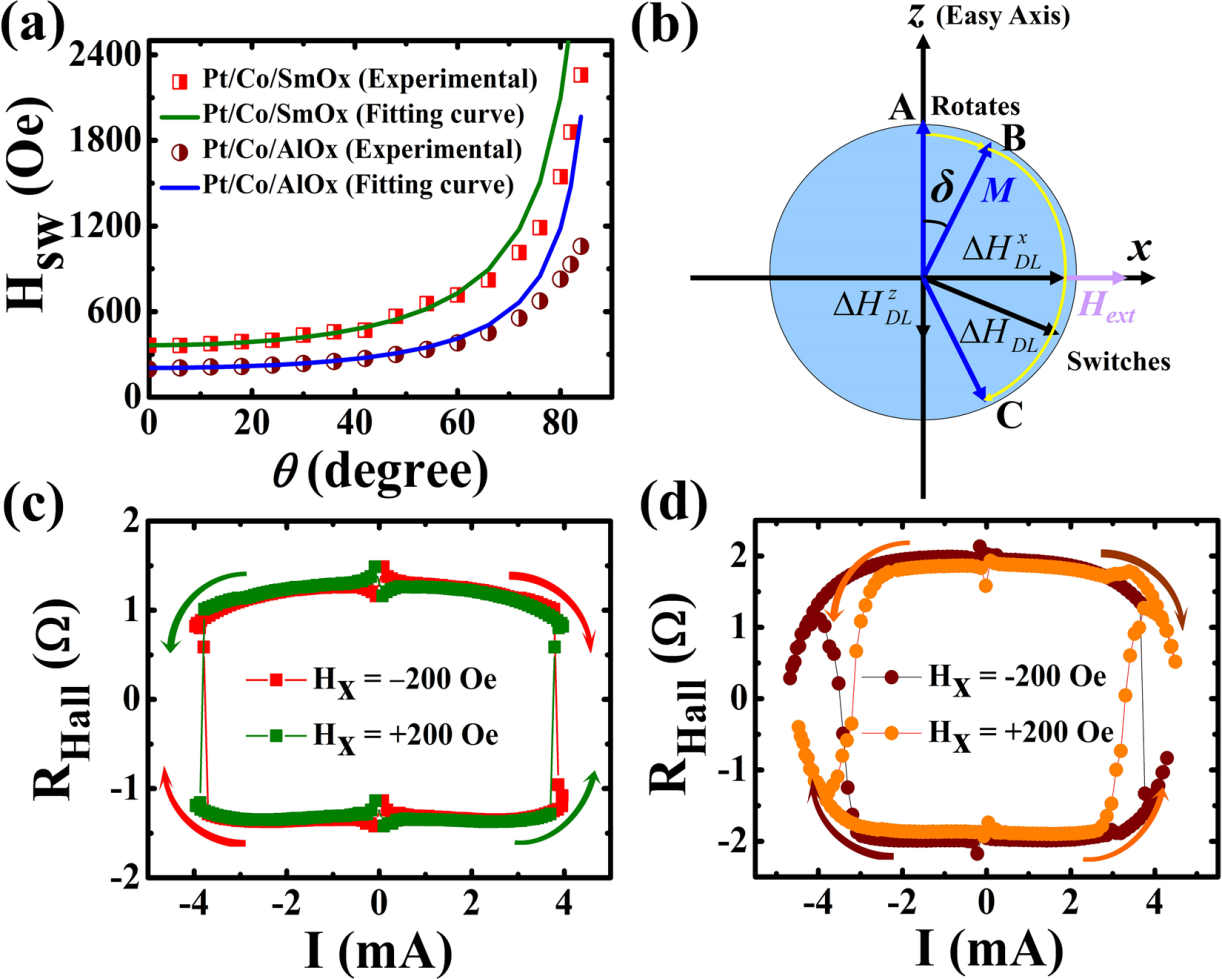
Current-induced magnetization reversal. (**a**) *H*_*sw*_ versus *θ. θ* is the angle between the *z* axis and *H*_*ext*_ in the *x*-*z* plane. (**b**) Schematic diagram illustrating the current-induced magnetization switching process. The perpendicular component ${\rm{\Delta }}{H}_{DL}^{x}$ = Δ*H*_*DL*_cos*δ* of Δ*H*_*DL*_ causes a rotation of the magnetization away from the easy axis, whereas the parallel component ${\rm{\Delta }}{H}_{DL}^{z}$ = Δ*H*_*DL*_sin*δ* is responsible for the irreversible switching. Current-induced magnetization reversal curves for Pt/Co/SmO_x_ (**c**) and Pt/Co/AlO_x_ (**d**) with an in-plane bias magnetic field of *H*_*x*_ = ± 200 Oe.

Combining the variation of the *H*_*sw*_ and Δ*H*_*DL*_ at different currents, we discuss the roles of the Joule heating and spin-orbit torque played in the current-induced magnetization reversal process. In Fig. 7(a), we characterize *H*_*sw*_-*θ* curves (*θ* is defined as the angle between the external magnetic field and *z* axis in the *x*-*z* plane) obtained from the angle dependent *R*_*Hall*_-*H*_*z*_ loops which are measured at *I* = 0.1 mA to eliminate the influence of the SOTs and Joule heating. From Fig. 7(a), one can see that *H*_*sw*_ follows an inverse cos*θ* relationship at small angles as shown in solid lines. It indicates that the magnetization reversal is dominated by the DW depinning in the large anisotropic systems (*H*_*sw*_ ≪ *H*_*k*_)^36,58,59^ when the external magnetic field deviates from the *z* axis in a small angle range. Figure 7(b) describes the schematic diagram of the current-induced magnetization reversal process. We assume that the magnetic moment vector (***M***) is rotated from A to B and deviates a small angle (*δ*) away from the *z* axis (easy axis) when applying an in-plane bias magnetic field (*H*_*ext*_). According to the equation which is expressed as Δ***H***_*DL*_ = Δ*H*_*DL*_(***σ*** × ***m***)^60^, where ***m*** and ***σ*** are the unit vector along the magnetization and spin polarization unit vector, respectively, the damping-like SOT induced effective field vector (Δ***H***_*DL*_) can be resolved into the perpendicular component (${\rm{\Delta }}{{\boldsymbol{H}}}_{DL}^{x}$) and parallel component (${\rm{\Delta }}{{\boldsymbol{H}}}_{DL}^{z}$). Here, Δ*H*_*DL*_ parameterizes the effective field, which can be written as Δ*H*_*DL*_ = *ħθ*_*SH*_*J*_*e*_/*2*|*e*|*M*_*s*_*t*_*FM*_. Thus, the ${\rm{\Delta }}{H}_{DL}^{x}$ = Δ*H*_*DL*_cos*δ* can further cause a rotation of the magnetization away from the easy axis, whereas the ${\rm{\Delta }}{H}_{DL}^{z}$ = Δ*H*_*DL*_sin*δ* is responsible for the irreversible magnetization reversal. Especially, ***M*** is switched from B to C when ${\rm{\Delta }}{H}_{DL}^{z}$ overcomes the depinning field.

Figure 7(c,d) show the current-induced magnetization reversal curves under an in-plane bias magnetic field of *H*_*x*_ = ±200 Oe for Pt/Co/SmO_x_ and Pt/Co/AlO_x_, respectively. According to ${M}_{Z}^{up}$ (${M}_{Z}^{down}$) corresponding to *R*_*Hall*_ < 0 (*R*_*Hall*_ > 0) in our experiment, the directions of the current and assisted *H*_*x*_ co-determine the polarity of the magnetization reversal. In addition, the critical switching current (*I*_*c*_) under the bias field of *H*_*x*_ = ±200 Oe is around 3.8 and 3.7 mA that correspond to the *J*_*c*_ of 4.4 × 10^6^ and 4.3 × 10^6^ A/cm^2^ for Pt/Co/SmO_x_ and Pt/Co/AlO_x_, respectively, assuming that the current uniformly flows across metallic layers. Since the magnitude of Δ*β*_*DL*_ for both samples is ~3.5 Oe per 10^6^ Acm^−2^ at RT as discussed above (see Supplementary Material S5 about Δ*β*_*DL*_ against *T*) and *J*_*c*_ is ~4.35 × 10^6^ Acm^−2^ for Pt/Co/SmO_x_ and Pt/Co/AlO_x_, the Δ*H*_*DL*_ is ~15 Oe and ${\rm{\Delta }}{H}_{DL}^{z}$ = Δ*H*_*DL*_sin*δ* may be more small at the critical switching process. Therefore, the ***M*** cannot have a switching only depending on the damping-like effective field. The current-induced magnetization reversal should be also on the basis of the contribution that the Joule heating from the current makes the *H*_*sw*_ decrease to a small value. That is, Joule heating plays an assisted role in the current-induced magnetization reversal and the out-of-plane component of Δ*H*_*DL*_ should be regarded as the effective driving force in overcoming the *H*_*sw*_. In addition, the results show that *J*_*c*_ remains roughly equivalent for Pt/Co/SmO_x_ and Pt/Co/AlO_x_ structures with similar spin Hall angles and different magnetic anisotropies, which can be also explained by that *H*_*sw*_ of the two stacks is reduced to a close value due to the Joule heating effect when the current reaches to *I*_*c*_. Finally, the weak *T* dependent *H*_*k*_ (see the Supplementary Material S5) has also demonstrated that *H*_*k*_ has no substantial influence on the depinning mechanism with the existence of Joule heating. Whereas, the tendency of *H*_*sw*_ varying versus *I* or *T* may be related to *H*_*k*_ in the thermal assisted magnetization reversal. Besides, the *T* dependent *M*_*s*_ and Δ*β*_*DL*_ (see the Supplementary Material S5) reveal that the current-generated Joule heating can also enhance SOT efficiency like *T*.

Based on above discussions, it also gives us some enlightenment on the current induced field-free magnetization reversal. On one hand, Δ*H*_*DL*_ is along the *x* axis (*δ* = 0) without an in-plane bias field *H*_*ext*_. When increasing the current, the Δ*H*_*DL*_ is also increasing, which can make ***M*** rotating away from *z* axis (*δ* > 0). ${\rm{\Delta }}{H}_{DL}^{z}$ = Δ*H*_*DL*_sin*δ* which is responsible for the irreversible switching yields at the same time. On the other hand, the Joule heating is gradually predominant as the current increases, which can dramatically decrease the *H*_*sw*_. Therefore, ***M*** can be switched when the competition of ${\rm{\Delta }}{H}_{DL}^{z}$ and *H*_*sw*_ is comparable at a certain current without the assistance of an in-plane bias field. However, the larger switching current induced thermal fluctuation may destroy the thermal stability of devices and even the devices may be burned at the large current. It is essential to choose the materials and structures with the larger PMA and/or *θ*_*SH*_, because the former can resist the thermal fluctuation from Joule heating and the latter can reduce the switching current and Joule heating.

## Conclusions

In summary, we have investigated the roles of the Joule heating and current-induced spin-orbit torque played in the current-induced magnetization reversal in Pt/Co/SmO_x_ and Pt/Co/AlO_x_ structures with different perpendicular magnetic anisotropies. The results show that the Joule heating generated from the direct current plays an assisted role in the switching process since it dramatically decreases the switching field and enhances the SOT efficiency. The out-of-plane component of the damping-like-SOT effective field is the real effective field which drives magnetic moments switching. When the Joule heating makes the switching field reduce to a critical threshold that the effective field can overcome, the magnetization realizes a full reversal. Moreover, the switching current density shows no prominent difference for Pt/Co/SmO_x_ and Pt/Co/AlO_x_ structures with similar spin Hall angles and different magnetic anisotropies, implying that the coherent switching model cannot fully explain the current-induced magnetization reversal. Nevertheless, by observing the domain wall nucleation and expansion during the field-induced magnetization reversal, we conclude that the depinning model should be dominant and the switching current density also relies on the switching field (i.e. depinning field) which can be greatly affected by Joule heating. The observed non-linear current dependence of the field-like-SOT effective field in both structures may be also affected by Joule heating. These findings could provide a legible picture of the SOT-induced magnetization reversal and highlight the assistant role of the current-generated Joule heating, which is conducive to understand and manipulate current-induced magnetization reversal in SOT-based spintronic devices.

## Methods

### Sample preparation

The stacks with the structures of Ta(3)/Pt(5)/Co(0.6)/SmO_x_(1)/Ta(3) and Ta(3)/Pt(5)/Co(0.6)/AlO_x_(0.5)/Ta(3) (thickness in nm) were deposited on corning glass substrates with the thickness of 500 µm by direct current (dc) magnetron sputtering. The growth was carried out at room temperature with a base pressure below 4.0 × 10^−5^ Pa. Among the samples, the bottom 3 nm Ta is used as the seed layer to enhance the PMA and the top 3 nm Ta as capping layer in order to prevent degradation of the oxide layers due to air exposure and the annealing. The thin film stacks were patterned into Hall bars with the width of 8.5 μm using standard photo-lithography and Ar-ion milling. The Hall bars were connected by Ta(20 nm)/Pt(30 nm) electrode pads. Afterwards, the devices were annealed at 400 °C for 1 h with a background pressure of 3.0 × 10^−4^ Pa to obtain the PMA.

### Measurement setup

The anomalous Hall resistances were measured using the electric transport measurements with a Keithley 6221 current source for injecting a DC current into the Hall bar along the *x* axis and a 2182 nano-voltmeter for collecting the Hall voltage across the bar along the *y* axis (Fig. 1(a)). The harmonic Hall voltage measurements were carried out using a sinusoidal current with a frequency of 133 Hz generated by the Keithley 6221 current source and the harmonic voltages were collected using an Analog-Digital (AD)/Digital-Analog (DA) data acquisition card. The saturation magnetization and planar Hall resistance were determined with a physical property measurement system (PPMS). A polar magneto-optical Kerr microscope was used to record the polar Kerr hysteresis loops and Kerr differential images of the domain wall nucleation and expansion during the field-induced magnetization switching. In order to obtain the Kerr differential images, the film was firstly saturated in one direction using a large out-of-plane magnetic field and then the saturated domain was chose as the background. After subtracting the background, the nucleated domain in differential modes appeared by applying a reverse magnetic field. The cross region in the middle of the Hall bar (Fig. 2(a,b)) was served as the measured area to collect the Kerr hysteresis loops and determine the switching field.

## Electronic supplementary material

Supplementary Material

## Data Availability

The datasets generated during the current study are available from the corresponding author on reasonable request.
