# Supplementary material for: Roles of Joule heating and spin-orbit torques in the direct current induced magnetization reversal
Source: Sci Rep. 2018 Aug 28;8:12959. doi: 10.1038/s41598-018-31201-2 (PMC6113327; doi:10.1038/s41598-018-31201-2)
Supplement: Supplementary file 1 — Supplementary Material [file 41598_2018_31201_MOESM1_ESM.pdf]

Roles of Joule heating and spin-orbit torques in the direct current induced magnetization reversal

Dong Li†, ‡, Shiwei Chen†, Yalu Zuo†, Jijun Yun†, Baoshan Cui†, Kai Wu†, Xiaobin Guo†, Dezheng Yang†, Jianbo Wang†, & Li Xi†

SUPPLEMENTARY INFORMATION

Contents:

S1. The demonstration of the direct current generated Joule heating.

S2. Polar Kerr hysteresis loops and Kerr differential images of the domain wall nucleation and expansion measured at different temperatures.

S3. Polar Kerr hysteresis loops and Kerr differential images of the domain wall nucleation and expansion measured at different pulse voltages.

S4. Measurements of the planar Hall resistance as well as the correlation between current induced spin-orbit effective fields and I_0 before the planar Hall effect correction.

S5. The temperature dependence of the anisotropy field (H_k), saturated magnetization (M_s) and damping-like effective field per unit current density ($\Delta H_{DL}/J_e$).

†Key Laboratory for Magnetism and Magnetic Materials of Ministry of Education & School of Physical Science and Technology, Lanzhou University, Lanzhou 730000, People's Republic of China. ‡Research Institute of Materials Science, Shanxi Normal University, Linfen 041004, People's Republic of China. Correspondence and requests for materials should be addressed to Li Xi (email: xili@lzu.edu.cn)

S1. The demonstration of the direct current generated Joule heating.

In order to demonstrate the existence of the direct current generated Joule heating, the dependence of the longitudinal voltage (V_{xx}) on the current (I) for Pt/Co/AlO_x sample is measured using the four-point method shown in Fig. S1 (a). Fig. S1 (b) shows the corresponding dependence of the longitudinal resistance (R_{xx}) on the current. The nonlinear dependence indicates the existence of Joule heating. For a quantitative study of the Joule heating, we also measure the temperature (T) dependence of R_{xx} in a range from 100 K to 400 K at a small current of 0.1 mA. By the linear fitting, the quantitative relationship of R_{xx} and T can be obtained. Comparing these two sets of data (R_{xx}~T and R_{xx}~I), we can estimate the temperature changes at different direct currents, which is shown in Fig. S1 (d).

S2. Polar Kerr hysteresis loops and Kerr differential images of the domain wall nucleation and expansion measured at different temperatures.

In order to reveal the effect of the Joule heating ($Q_J \sim I^2$) generated from the direct current on the field-induced magnetization reversal as well as DW nucleation and expansion behavior, we investigate the variation of the polar Kerr hysteresis loops and Kerr differential images at different T without applying any currents. Fig. S2 (a) and (b) show the polar Kerr hysteresis loops at various T for Pt/Co/SmO_x and Pt/Co/AlO_x, respectively. Similarly, from Fig. S2 (a) and (b), one can see that the loops gradually become narrow as T increases for Pt/Co/SmO_x and Pt/Co/AlO_x, indicating the decrease of H_{sw} with T increasing. In addition, the Kerr differential images of the domain wall nucleation and expansion were also observed when collecting the Kerr hysteresis loops. Fig. S2 (c) and (d) show representative Kerr differential images of the DW nucleation and expansion measured at different T for Pt/Co/SmO_x and Pt/Co/AlO_x, respectively. The light (deep) color in the images corresponds to the "up" ("down") domain or magnetized state. As the same as the case at the direct currents described in Fig. 3 of the main text, we can observe that both the nucleation field and switching field in the measured regions gradually decrease as T increases for the two stacks. The high temperature can also change the nucleation site and expansion speed, revealing that the temperature can affect the H_{sw} by DW nucleation and expansion. It is also noted that the resolution of images at the higher temperature reduces due to that the temperature changes the optimized focusing height of samples.

S3. Polar Kerr hysteresis loops and Kerr differential images of the domain wall nucleation and expansion measured at different pulse voltages.

In order to investigate the effect of the current-induced SOT ($\tau \sim I$) on the field-induced magnetization reversal as well as DW nucleation and expansion behavior, we explore the variation of the polar Kerr hysteresis loops and Kerr differential images at different pulse voltages (V_p) using a 300 picosecond pulse generator (Tektronix: PSPL10300B) by fading out the Joule heating effect. Fig. S3 (a) and (b) depict polar Kerr hysteresis loops of Pt/Co/SmO_x and Pt/Co/AlO_x stacks measured at different V_p with the fixed pulse period (T_p) of 10 μs and pulse width (W_p) of 100 ns. It is noted that the V_p of 28.1 V (14.1 V) corresponds to the pulse current (I_p) of about 4.5 mA (4mA) for Pt/Co/SmO_x (Pt/Co/AlO_x). In Fig. S3 (a) and (b), one can see that the loops are still wide at large V_p compared to that at the direct currents as discussed in Fig. 2 (c) and (d) of the main text. Whereas, when increasing the T_p to 1 ms for Pt/Co/SmO_x stacks and 100 μs for Pt/Co/AlO_x as shown in Fig. S3 (c) and (d), the loops are nearly coincided at different V_p (the current-generated Joule heating can be negligible), indicating that the effect of the current-induced SOT on the magnetization reversal is rather weak compared to the influence of the temperature or Joule heating. As depicted in Fig. S3 (e) and (f), the Kerr images of the DW nucleation and expansion measured at different V_p also show no difference with each other for Pt/Co/SmO_x with T_p=1 ms and W_p=100 ns and Pt/Co/AlO_x with T_p=100 μs and W_p=100 ns, respectively.

S4. Measurements of the planar Hall resistance as well as the correlation between current induced spin-orbit effective fields and I_0 before the planar Hall effect correction.

To measure the planar Hall resistance (ΔR_{PHE}), a large in-plane magnetic field of 5 T was applied to fully make the magnetization in the *x*-*y* plane. And then, the Hall resistance (R_{Hall}) was recorded as a function of the in-plane angle φ (the angle is shown in the inset of Fig. S4 (a)). Fig. S4 (a) and (b) show the variation of R_{Hall} against φ for Pt/Co/SmO_x and Pt/Co/AlO_x, respectively. The ΔR_{PHE} is defined as the half of the difference between the maximum and minimum values of R_{Hall}, which is depicted in Fig. S4 (a). Therefore, ΔR_{PHE} is obtained which equals to 0.26 and 0.29 Ω for Pt/Co/SmO_x and Pt/Co/AlO_x stacks, respectively. The anomalous Hall resistance (ΔR_{AHE}) with the same definition as ΔR_{PHE} can also be obtained by the measurement shown in Fig. 1 (b) and (c) of

the main text. The corresponding ξ (= $\Delta R_{PHE}/\Delta R_{AHE}$) is found to be 0.15 for both the stacks.

Before the planar Hall effect (PHE) correction, the calculated damping-like effective field (H_{DL}) and field-like (H_{FL}) effective field using Eq. (2) in the main text as a function of the amplitude (I_0) of the sinusoidal current are shown in Fig. S4 (c)-(f). Fig. S4 (c) and (d) show H_{DL} against I_0 for Pt/Co/SmO$_x$ and Pt/Co/AlO$_x$ stacks, respectively, which presents a roughly linear relationship. Thus, H_{DL} per unit current density (β_{DL}) can be found to be -3.67 ± 0.02 Oe/(10^6 A/cm^2) (2.40 ± 0.12 Oe/(10^6 A/cm^2)) for the "up" ("down") magnetized state for Pt/Co/SmO$_x$ and -3.30 ± 0.12 Oe/(10^6 A/cm^2) (3.35 ± 0.13 Oe/(10^6 A/cm^2)) for the "up" ("down") magnetized state for Pt/Co/AlO$_x$ using $\beta_{DL}=H_{DL}/J_e$ (J_e is the charge current density). It is evident that the damping-like SOT induced effective field is almost equivalent, which is due to the same contribution from the heavy metal Pt. Besides, the H_{FL} is also plotted as a function of I_0 in the same manner for both devices displayed in Fig. S4 (e) and (f). Unlike H_{DL}, a non-linear behavior of H_{FL} versus I_0 is observed, which behaves like a quadratic dependence. The corrected H_{DL} and H_{FL} by considering PHE are shown in Fig. 6 of the main text using the equation below:[1]

$$\Delta H_{DL(FL)} = \frac{H_{DL(FL)} \pm 2\xi H_{FL(DL)}}{1-4\xi^2}.$$

where the ± sign refers to the "up" and "down" magnetized states and $\xi = \Delta R_{PHE}/\Delta R_{AHE}$.

S5. The temperature dependence of the anisotropy field (H_k), saturated magnetization (M_s) and damping-like effective field per unit current density ($\Delta H_{DL}/J_e$).

(A) The temperature dependence of the anisotropy field (H_k).

In order to exclude the effect of the anisotropy field (H_k) on the depinning mechanism due to the existence of Joule heating, we investigate the H_k as a function of T in a range from 100 K to 400 K for Pt/Co/SmO$_x$ and Pt/Co/AlO$_x$ samples. The H_k is determined by measuring R_{Hall} versus the in-plane field (H_x) loops at different temperatures using the Eq. (1) in the main text. As shown in Fig. S5 (a) and (b), the H_k decreases weakly as T increases for Pt/Co/SmO$_x$, especially the H_k still keeps a large value (≈6000 Oe) at 400 K and the H_k remains roughly constant for Pt/Co/AlO$_x$ at different T. Meanwhile, we also measures H_k against T for other Pt/Co/SmO$_x$ and Pt/Co/AlO$_x$ bars in the batch, which is shown in Fig. S5 (c) and (d). Although the size of H_k shows some

differences, the tendency of H_k against T remains similar, indicating that H_k indeed presents a weak temperature dependence.

In addition, R_{Hall}-H_z loops are measured at the large current (large Joule heating) for Pt/Co/SmO_x and Pt/Co/AlO_x samples shown in Fig. S6 (a) and (b). They both still keep the sharp square-shaped loops and relatively large R_{Hall}, confirming the presence of well PMA at large Joule heating. By contrast, the switching field becomes very small for both samples. Therefore, the depinning model should be dominant.

(B) The temperature dependence of the saturated magnetization (M_s) and damping-like effective field per unit current density ($\Delta H_{DL}/J_e$).

Fig. S7 (c) and (d) show the T dependence of M_s for Pt/Co/SmO_x and Pt/Co/AlO_x, and Fig. S7 (a) and (b) illustrate the representative magnetic hysteresis loops at different temperatures measured by the physical property measurement system (PPMS). One can see that the M_s decreases gradually as the T increases for both Pt/Co/SmO_x and Pt/Co/AlO_x samples. In SHE picture, the spin-orbit torque efficiency is determined by the damping-like effective field per unit current density which is related to the size of M_s according to the formula $\Delta H_{DL}/J_e=\hbar\theta_{SH}/2eM_st_{FM}$[2, 3] (where the ΔH_{DL} is the damping-like effective field, J_e is the charge current density, and θ_{SH} is the spin Hall angle). When the T gradually increases, M_s decreases, which may make $\Delta H_{DL}/J_e$ increasing. The T dependence of $\Delta H_{DL}/J_e$ shown in Fig. S8 (a) and (b) for Pt/Co/SmO_x and Pt/Co/AlO_x samples also demonstrates that the $\Delta H_{DL}/J_e$ increases as T increases gradually. Besides, the dependence of ΔH_{DL} on the amplitude (I_0) of the sinusoidal current, especially the ΔH_{DL} deviating the roughly linear fitting at the large I_0, shown in Fig. 6 (a) and (c) in the main text also demonstrates the influence of Joule heating. Although the Joule heating generated from the sinusoidal current is smaller than that from the direct current at the same current amplitude, the Joule heating becomes also non-negligible at the large I_0, which can lead to the ΔH_{DL} deviating the linear relationship at the I_0 of 3 mA. The Joule heating effect is considered to be a non-linear behavior in the I_0 dependence of ΔH_{DL} as reported in the previous work.[4, 5] Therefore, the decreasing M_s makes the $\Delta H_{DL}/J_e$ increasing. However, the $\Delta H_{DL}/J_e$ is still relatively small and does not change by the order of the magnitude, even though the $\Delta H_{DL}/J_e$ increases as T or Joule heating increases gradually. For instance, the $\Delta H_{DL}/J_e$ at 300 K is -3.58 ± 0.19 Oe/(10^6 A/cm^2)

(3.29 ± 0.19 Oe/(10^6 A/cm^2)) for the "up" ("down") magnetized state for Pt/Co/SmO_x and -3.64 ± 0.07 Oe/(10^6 A/cm^2) (3.91 ± 0.09 Oe/(10^6 A/cm^2)) for the "up" ("down") magnetized state for Pt/Co/AlO_x. However, the $\Delta H_{DL}/J_e$ is -4.27 ± 0.17 Oe/(10^6 A/cm^2) (3.91 ± 0.24 Oe/(10^6 A/cm^2)) for the "up" ("down") magnetized state for Pt/Co/SmO_x and -4.04 ± 0.15 Oe/(10^6 A/cm^2) (4.42 ± 0.13 Oe/(10^6 A/cm^2)) for the "up" ("down") magnetized state for Pt/Co/AlO_x when the T increases to 400 K. It means that the magnitude of $\Delta H_{DL}/J_e$ for both samples is still a small value at higher T. Whereas, J_c is ~ 4.35×10^6 Acm^{-2} for Pt/Co/SmO_x and Pt/Co/AlO_x, the ΔH_{DL} is estimated to be small and $\Delta H^z_{DL}=\Delta H_{DL}\sin\delta$ may be smaller, indicating that ΔH_{DL} is also still a relatively small value so that it cannot overcome the large anisotropy field according to the coherent switching model. As a consequence, the switching mechanism should be the domain wall nucleation and expansion based on a depinning model.

References

1. Hayashi, M. *et al*. Quantitative characterization of the spin-orbit torque using harmonic Hall voltage measurements. *Phys. Rev. B* **89**, 144425 (2014).

2. Liu, L., Lee *et al*. Current-induced switching of perpendicularly magnetized magnetic layers using spin torque from the spin Hall effect. *Phys. Rev. Lett.* **109**, 096602 (2012).

3. Hao, Q. & Xiao, G. Giant spin Hall effect and magnetotransport in a Ta/CoFeB/MgO layered structure: A temperature dependence study. *Phys. Rev. B* **91**, 224413 (2015).

4. Qiu, X. P. *et al*. Angular and temperature dependence of current induced spin-orbit effective fields in Ta/CoFeB/MgO nanowires (see Supplementary Information). *Sci. Rep.* **4**, 4491 (2014).

5. Wu, D. *et al*. Spin-orbit torques in perpendicularly magnetized $Ir_{22}Mn_{78}/Co_{20}Fe_{60}B_{20}$/MgO multilayer. *Appl. Phys. Lett*. **109**, 222401 (2016).

Figures

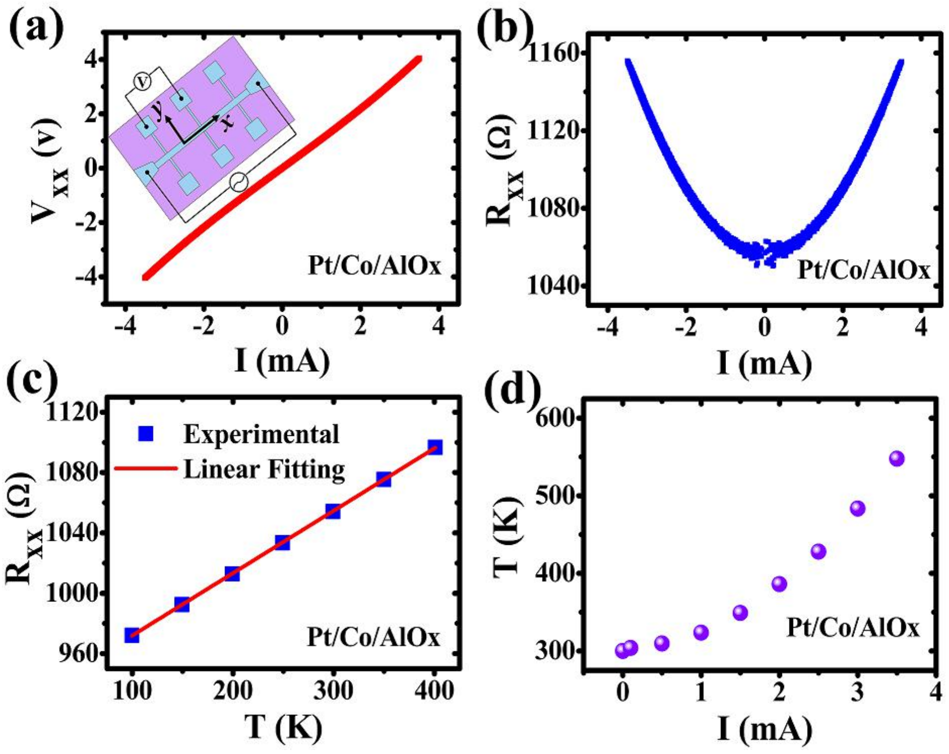

Figure S1. Current-generated Joule heating. (a) The dependence of longitudinal voltage (V_{xx}) on the current for Pt/Co/AlO_x samples measured by the four-point method, demonstrating the existence of Joule heating. The inset is the measurement diagram. **(b)** The dependence of longitudinal resistance (R_{xx}) on the current. **(c)** The longitudinal resistance (R_{xx}) as a function of the temperature. **(d)** Temperature changes at different direct currents.

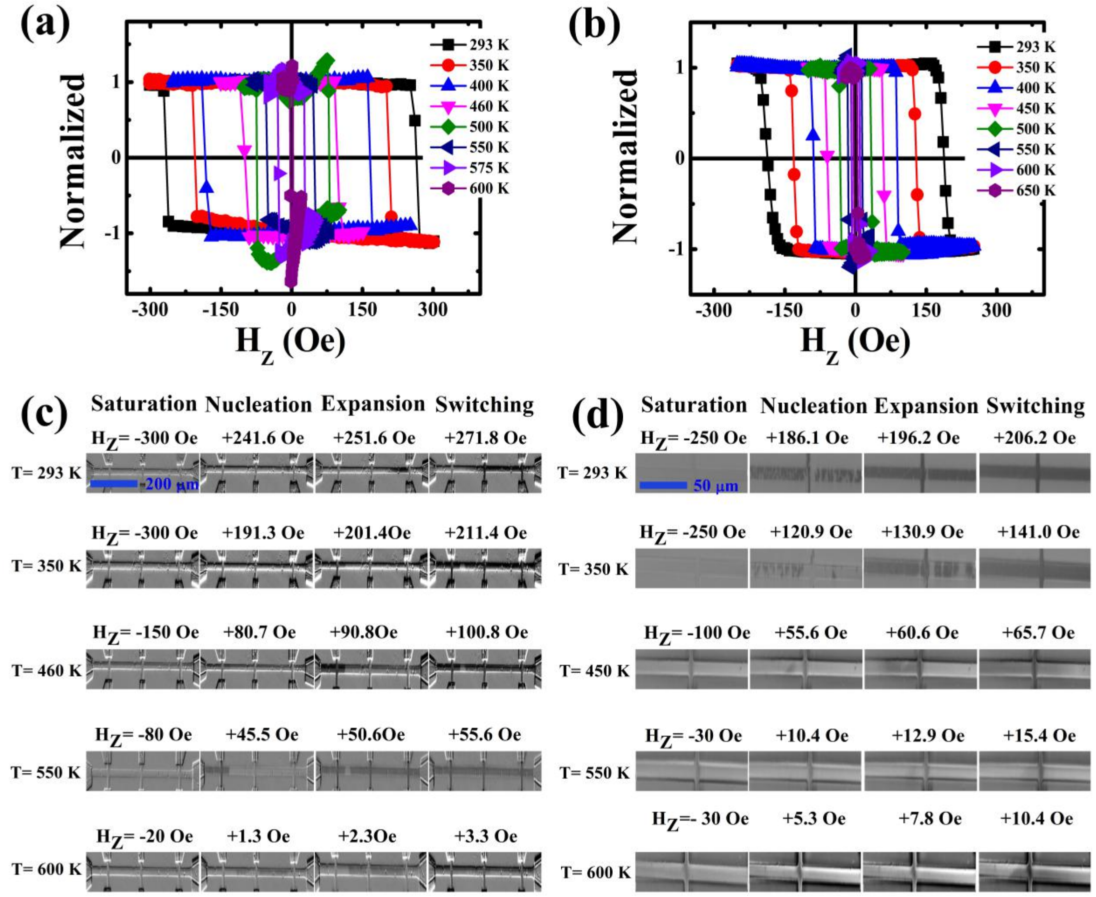

Figure S2. Polar Kerr hysteresis loops and Kerr differential images at different temperatures. Polar Kerr hysteresis loops for Pt/Co/SmO_x **(a)** and Pt/Co/AlO_x **(b)** stacks measured at different temperatures. Representative Kerr images for Pt/Co/SmO_x **(c)** and Pt/Co/AlO_x **(d)** during the field-induced magnetization switching at various temperatures, showing the effect of the temperature on DW nucleation and expansion. The light (deep) color shows the "up" ("down") magnetized state.

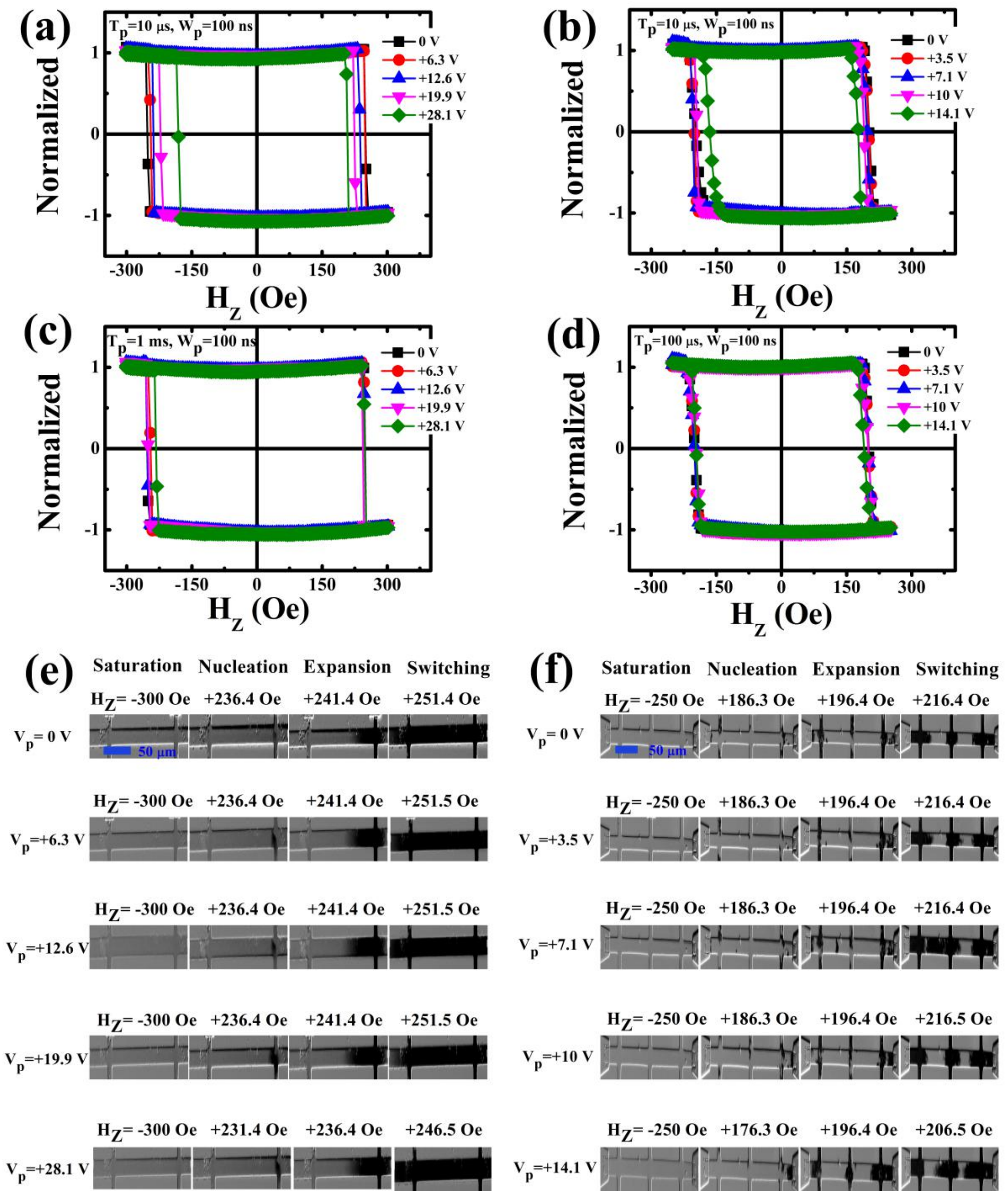

Figure S3. Polar Kerr hysteresis loops and Kerr differential images at different pulse voltages. Polar Kerr hysteresis loops for Pt/Co/SmO_x **(a)** and Pt/Co/AlO_x **(b)** stacks measured at different pulse voltages with the fixed pulse period T_p=10 μs and pulse width W_p=100 ns. Polar Kerr hysteresis loops for Pt/Co/SmO_x **(c)** with T_p=1 ms and W_p=100 ns and Pt/Co/AlO_x **(d)** with T_p=100 μs and W_p=100 ns measured at different pulse voltages. Representative Kerr images for Pt/Co/SmO_x **(e)** and Pt/Co/AlO_x **(f)** during the field-induced magnetization switching at various pulse voltages, showing the effect of the pulse voltage on DW nucleation and expansion. The light (deep) color shows the “up” (“down”) magnetized state.

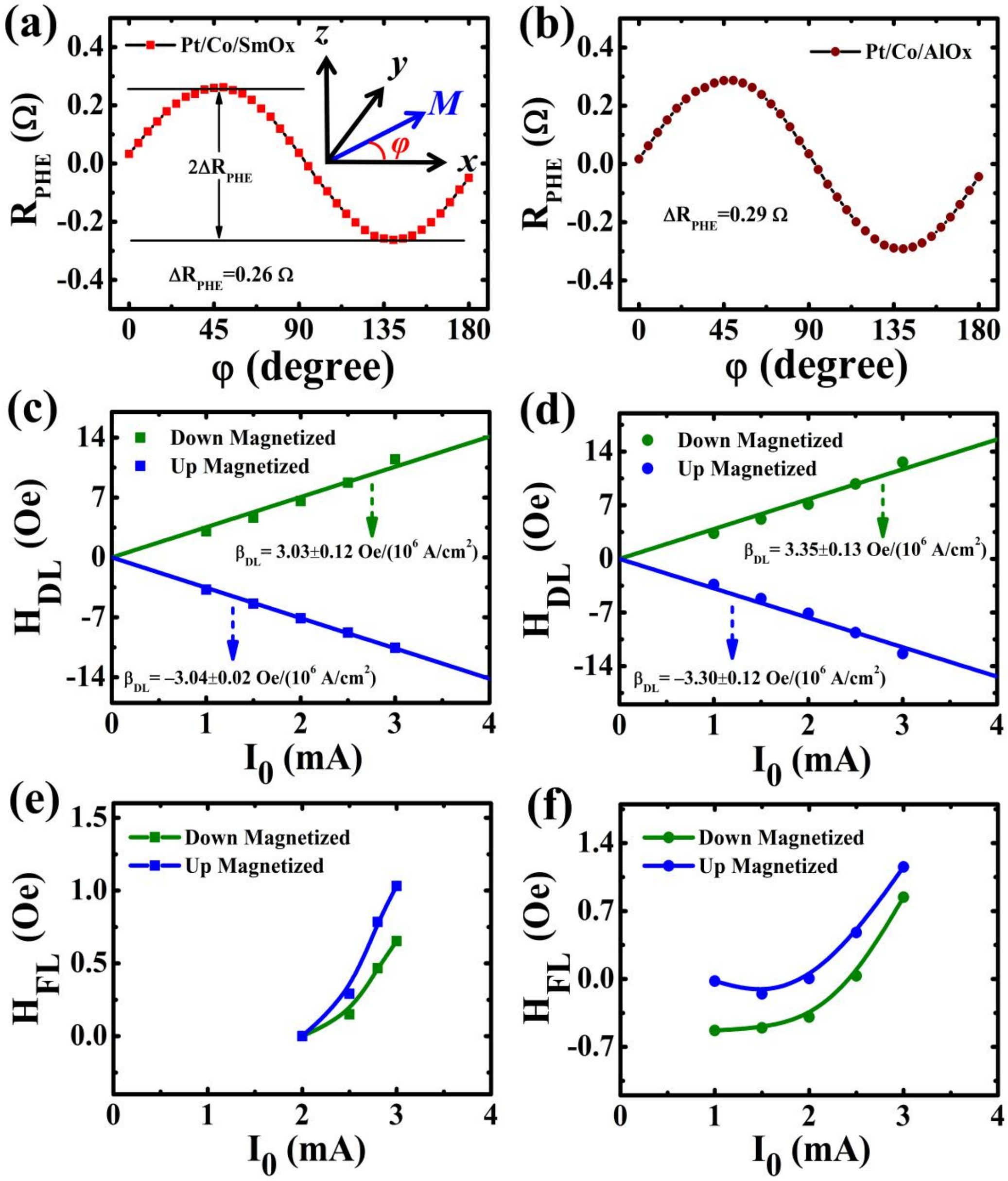

Figure S4. Current-induced effective fields before the planar Hall effect correction. Planar Hall resistance R_{PHE} as a function of the angle φ for Pt/Co/SmO$_x$ **(a)** and Pt/Co/AlO$_x$ **(b)**, respectively. φ is the angle between the magnetic moment M and x axis in x-y plane as shown in the inset. **(c)** and **(e)** (**(d)** and **(f)**) SOT-induced effective field for Pt/Co/SmO$_x$ (Pt/Co/AlO$_x$) against the amplitude I_0 of the sinusoidal current before the planar Hall effect correction. The blue and green symbols represent "up" and "down" magnetized states, respectively.

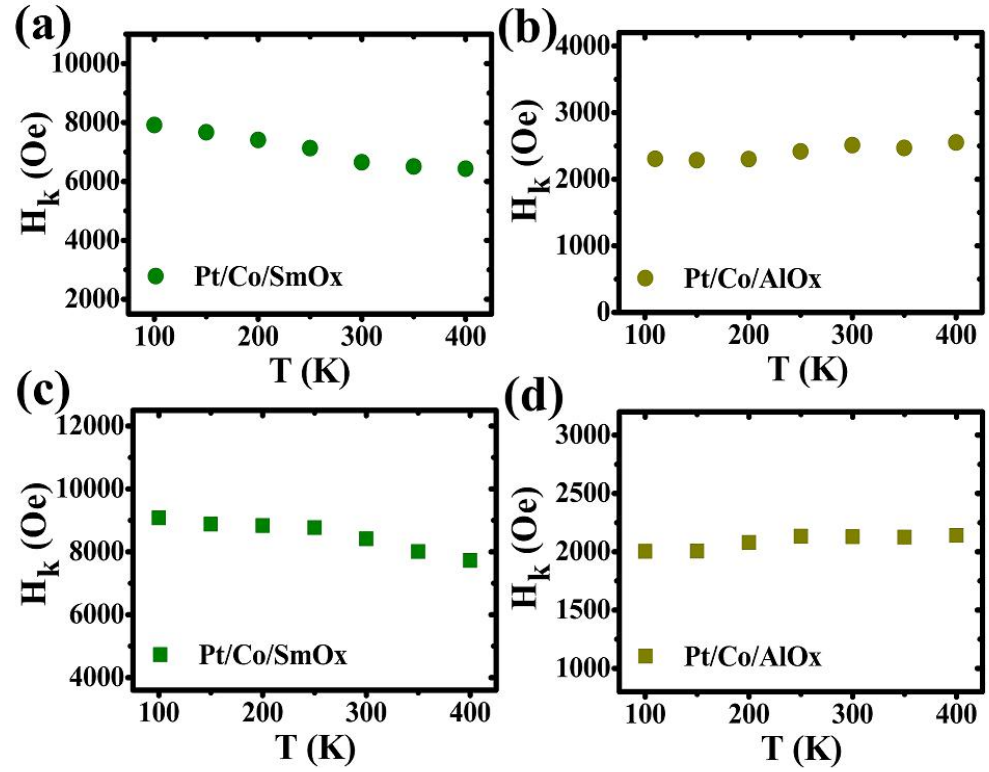

Figure S5. The temperature dependence of the anisotropy fields. The anisotropy fields (H_k) as a function of temperature (T) for **(a)** Pt/Co/SmO_x, and **(b)** Pt/Co/AlO_x samples. **(c)** and **(d)** show the H_k versus T for Pt/Co/SmO_x and Pt/Co/AlO_x samples, respectively, which are measured using other bars in the batch.

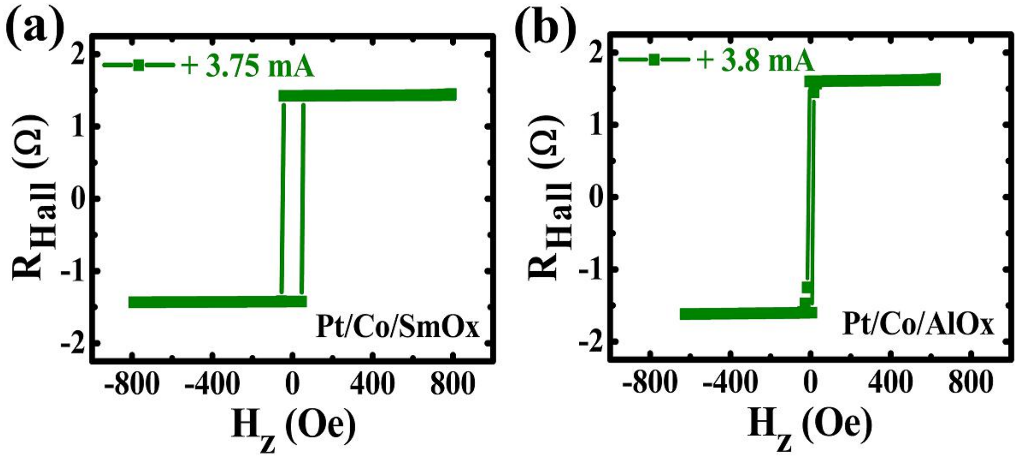

Figure S6. Anomalous Hall loops at large currents. R_{Hall}-H_z loops measured at + 3.75 mA and + 3.8 mA for **(a)** Pt/Co/SmO_x and **(b)** Pt/Co/AlO_x samples, respectively.

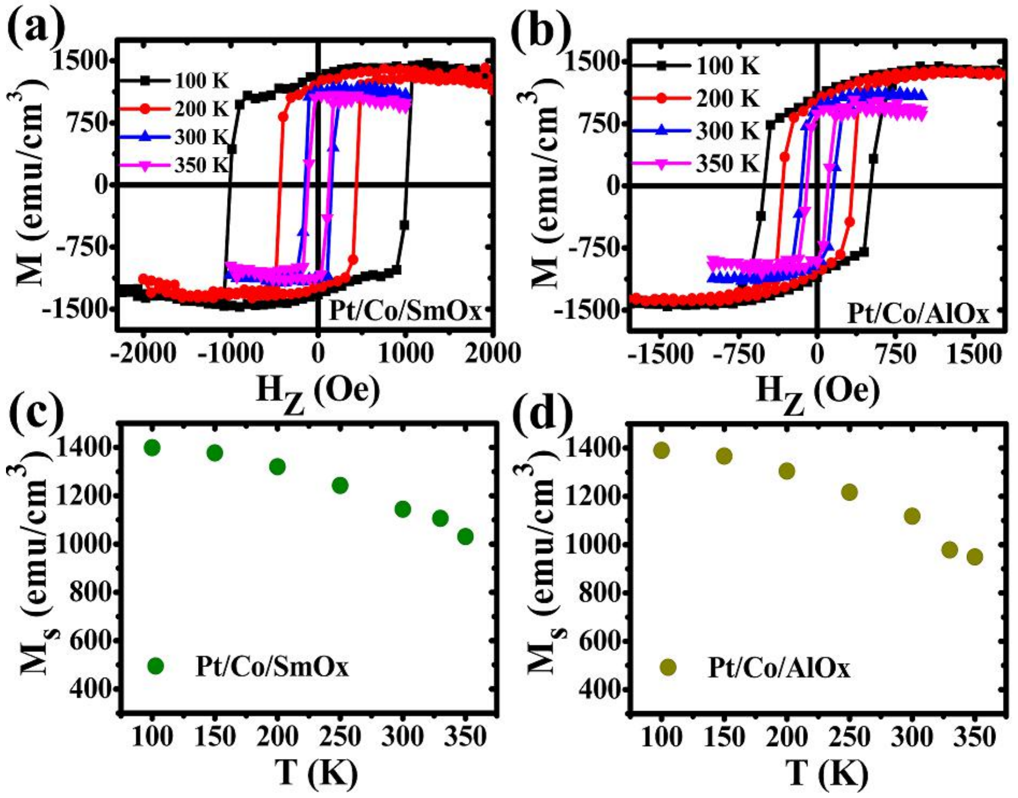

Figure S7. The temperature dependence of the saturated magnetization. Magnetic hysteresis loops at different temperatures for **(a)** Pt/Co/SmO$_x$ and **(b)** Pt/Co/AlO$_x$ samples. The saturated magnetization (M_s) as a function of the temperature (T) for **(c)** Pt/Co/SmO$_x$ and **(d)** Pt/Co/AlO$_x$ samples.

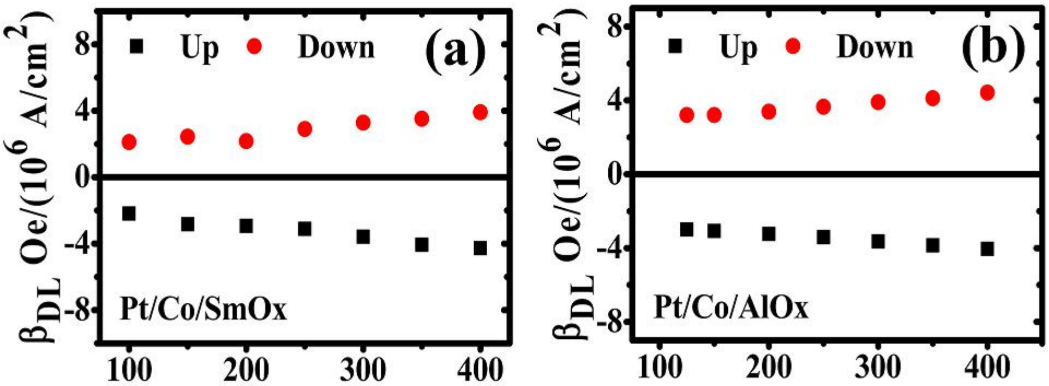

Figure S8. The temperature dependence of the damping-like SOT efficiency. β_{DL} ($=\Delta H_{DL}/J_e$) as a function of T for **(a)** Pt/Co/SmO$_x$ and **(b)** Pt/Co/AlO$_x$.
